# Efficacy and safety of different drugs for the treatment of bacterial vaginosis: a systematic review and network meta-analysis

**DOI:** 10.3389/fcimb.2024.1402346

**Published:** 2024-10-11

**Authors:** Yuxin Fan, Yanhong Gu, Yi Xian, Qinya Li, Youli He, Kaiyang Chen, Hui Yu, Huan Deng, Li Xiong, Zhiwei Cui, Yang Yang, Yin Xiang

**Affiliations:** Laboratory Department, The People’s Hospital of Leshan, Leshan, China

**Keywords:** bacterial vaginosis, treatment, antibiotic, sucrose, network meta-analysis

## Abstract

**Objective:**

Bacterial vaginosis is a disease caused by vaginal microecology disorder, which seriously affects female health. At present, there are many drugs to treat BV, and this study aims to compare the efficacy and safety of multiple drugs for BV through a network meta-analysis (NMA).

**Methods:**

All studies were sourced from PubMed and Embase databases from the establishment date to April 13, 2023. We evaluated the clinical cure success rate and adverse effects (abnormal increase in vaginal discharge, external genital irritation, and vulvar itching) and performed subgroup analyses of the clinical cure success rate for different modes of administration. All statistical analyses were performed using R and STATA 14.0 software for network meta-analysis.

**Results:**

We included 42 studies that met the criteria, involving a total of 8382 patients. Network meta-analysis results showed that metronidazole and secnidazole had a higher rate of adverse reactions than placebo (RR 7.06; 95%-CI 2.61-19.10, RR 4.03; 95%-CI 1.63-9.98), the adverse reaction rate of probiotics group was lower than that of metronidazole group (RR 0.44; 95%-CI 0.21-0.93). The clinical cure rate of oral ornidazole was better than clindamycin (RR 16.08; 95%-CI 1.72-150.47), Secnidazole (RR 8.17; 95%-CI 1.66-40.25) and probiotics. Direct meta-analysis results showed that ornidazole had a better clinical cure rate than Secnidazole (RR 1.22; 95%-CI 1.10-1.34), oral ornidazole had a better clinical cure rate than Secnidazole (RR 1.23; 95%-CI 1.11-1.36). The clinical cure rate of vaginal application of sucrose was better than metronidazole (RR 1.12; 95%-CI 1.03-1.21) and metronidazole had a lower clinical cure rate than probiotics (RR 0.68; 95%-CI 0.52-0.88).

**Conclusions:**

The results of this systematic review and network meta-analysis suggest that ornidazole may be an effective alternative for the treatment of BV, and that sucrose and probiotics are potential BV treatments that need to be validated by more high-quality clinical studies in the future.

## Introduction

Bacterial vaginosis (BV) is a disease caused by an imbalance of vaginal microecology (Coudray and Madhivanan, 2020). Vaginal flora is one of the most important defense mechanisms for reproductive function and maintaining a healthy environment. Healthy vaginal flora is composed of anaerobic bacteria and aerobic bacteria, with Lactobacillus as the dominant microorganism, which can inhibit the growth and colonization of pathogenic microorganisms through the production of lactic acid, H_2_O_2_, etc (Bautista et al., 2016; Ding et al., 2021). However, the environment of bacterial vaginitis has changed from a mixed environment dominated by lactobacilli to one of overgrowth of Gardnerella vaginalis and other anaerobic bacteria (e.g., *Atopobium vaginae*, *Bacteroides spp* and *Ureaplasma urealyticum*), and the clinical symptoms are generally vaginal discharge, vaginal odor, pruritus in the perineal area, and elevated vaginal pH (Smith and Ravel, 2017; Abou Chacra et al., 2021; Ding et al., 2021). The prevalence of BV ranges from 20-60% between different countries, with the highest prevalence in sub-Saharan Africa (Bautista et al., 2016).BV can increase the risk of contracting many sexually transmitted infections (STIs), such as human immunodeficiency virus (HIV), *Neisseria gonorrhoeae* (NG), Trichomonas vaginalis (TV), and herpes simplex virus-2 (HSV-2), among others (Gardella et al., 2017; Smith and Ravel, 2017; Paavonen and Brunham, 2020).In addition, BV is associated with adverse reproductive health outcomes such as preterm birth, miscarriage, infections, cervicitis, urethritis, salpingitis, and urinary tract infections, which significantly affect women’s health (Hillier et al., 1995; Leitich and Kiss, 2007; Baisley et al., 2009; Coudray and Madhivanan, 2020; Ravel et al., 2021).

In the 2015 guidelines for the management of sexually transmitted diseases (STDs), the Centers for Disease Control and Prevention (CDC) recommended BV treatment with metronidazole or clindamycin (Workowski and Bolan, 2015). By 2018, topical or oral metronidazole for 5-7 days or vaginal clindamycin for 7 days became the first-line treatment option for BV recommended by the World Health Organization (WHO) (Sherrard et al., 2018). Although the treatment is effective, excessive use of metronidazole and clindamycin can also cause side effects such as gastrointestinal reactions and allergic reactions (Menard, 2011; Ayinde and Ross, 2023). Several antibiotic drugs (tinidazole, metronidazole, ofloxacin, Secnidazole, etc.) and non-antibiotic drugs (sucrose, probiotics, etc.) are effective in the treatment of BV, and some of them have been or are in clinical phase I or Phase II randomized controlled trials (Donders et al., 2014). As an effective component of single small-molecule oligosaccharides, sucrose can selectively promote the growth of inherently beneficial lactobacilli in the vagina, lower vaginal pH, regulate the vaginal flora, and thus inhibit the growth of pathogenic bacteria (Lidbeck and Nord, 1993; Xiao et al., 2015).In addition, probiotic therapy is also considered to be one of the feasible ways to treat BV. However, there is currently no literature system evaluating their effectiveness and safety in the treatment of BV. This study selected eight alternative drugs for the treatment of BV and used a network meta-analysis (NMA) to compare efficacy and safety in the treatment of BV to determine which antibiotic was the best alternative to metronidazole.

NMA is a statistical technique that allows multiple treatments to be compared in the same meta-analysis and is based on meta-analysis techniques for weighted combined analysis. This approach allows for a combination of direct and indirect comparisons, where indirect comparisons can compare interventions that have not previously been compared in a randomized controlled trial, as well as comparing more than two interventions. And ranked in order of effect size to address differences in treatment effects among multiple complex interventions, providing an important reference for policymakers to develop clinical guidelines.

## Method

### Search strategy

We systematically searched potentially eligible studies in PubMed and EMBASE databases using a combination of medical subject title (MeSH) descriptors and free text terms (as of October 2023). Designed to search the following categories: “bacterial vaginitis”, “metronidazole”, “Clindamycin”, “Probiotics” and “placebo”. In addition, references for all included studies were manually searched and a meta-analysis was performed on the same topic to obtain additional eligible studies (see table in **Supplementary Materials**).

### Selection criteria

Articles included in this study must meet the following criteria: 1. Randomized controlled trial (RCT) or observational studies published in English; 2. The subjects were patients diagnosed with BV; 3. The effects of the treatment drugs for BV were compared with each other; 4. Primary outcomes included clinical cure rate and incidence of adverse reactions. In addition, reviews, editorials, conference abstracts, and case reports were excluded from the final dataset.

### Data extraction

For each included study, data extraction was performed by two independent examiners using standardized data extraction tables, and the relevant study characteristics extracted included: 1. The first author; 2. Year of publication; 3. Research type; 4. Disease type; 5. Basic information of the patient; 6. Type of treatment (antibiotics, metronidazole, probiotics, or placebo); 7. Medication information (medication method, dosage, and duration); 8. Prognosis and curative effect (clinical cure rate); 9. Adverse reactions.

### Outcome indicator

We considered two primary outcomes, clinical cure and adverse event (AE), in a network meta-analysis. Criteria for clinical cure include: (A) Vaginal pH ≤4.5; (B) Normal vaginal discharge, negative olfactory test; (C) Normal vaginal microbiota, the clue cells were less than 20% of the total epithelial cells in the saline tablet. Finally, safety, i.e. the incidence of adverse events, was assessed. An adverse event is defined as any adverse medical event associated with the use of a drug. The most common adverse reactions included abnormally increased vaginal discharge, enhanced vaginal odor, external genital irritation, and genital itching.

### Quality evaluation

The quality of the included studies was assessed by two independent reviewers based on the tools provided by the Cochrane Manual, and differences were resolved by two independent reviewers through discussion with a third reviewer, if necessary. Finally, Review Manager 5.4 software was used to generate the bias risk graph.

### Statistical analysis

Two outcome measures, clinical cure rate and incidence of adverse reactions, were evaluated by Bayesian network meta-analysis. In addition, a subgroup analysis of two different treatments in the clinical cure rate measure, oral and vaginal application, was performed to compare the effectiveness and safety of various drugs for the treatment of BV. Stata 14.0 software and R software were used for statistical analysis of the data. We analyzed all the data using a random effects model to obtain relative risk (RR) and 95% confidence intervals (95% CI), comparing the efficacy and safety of various drugs in a given population. A random-effects network meta-analysis was then performed, which was used to directly and indirectly compare the efficacy of various drugs. Funnel plots were drawn to evaluate publication bias in the included literature. This study is registered with PROSPERO, number CRD42023454041.

## Results

### Study selection

**Figure 1:**
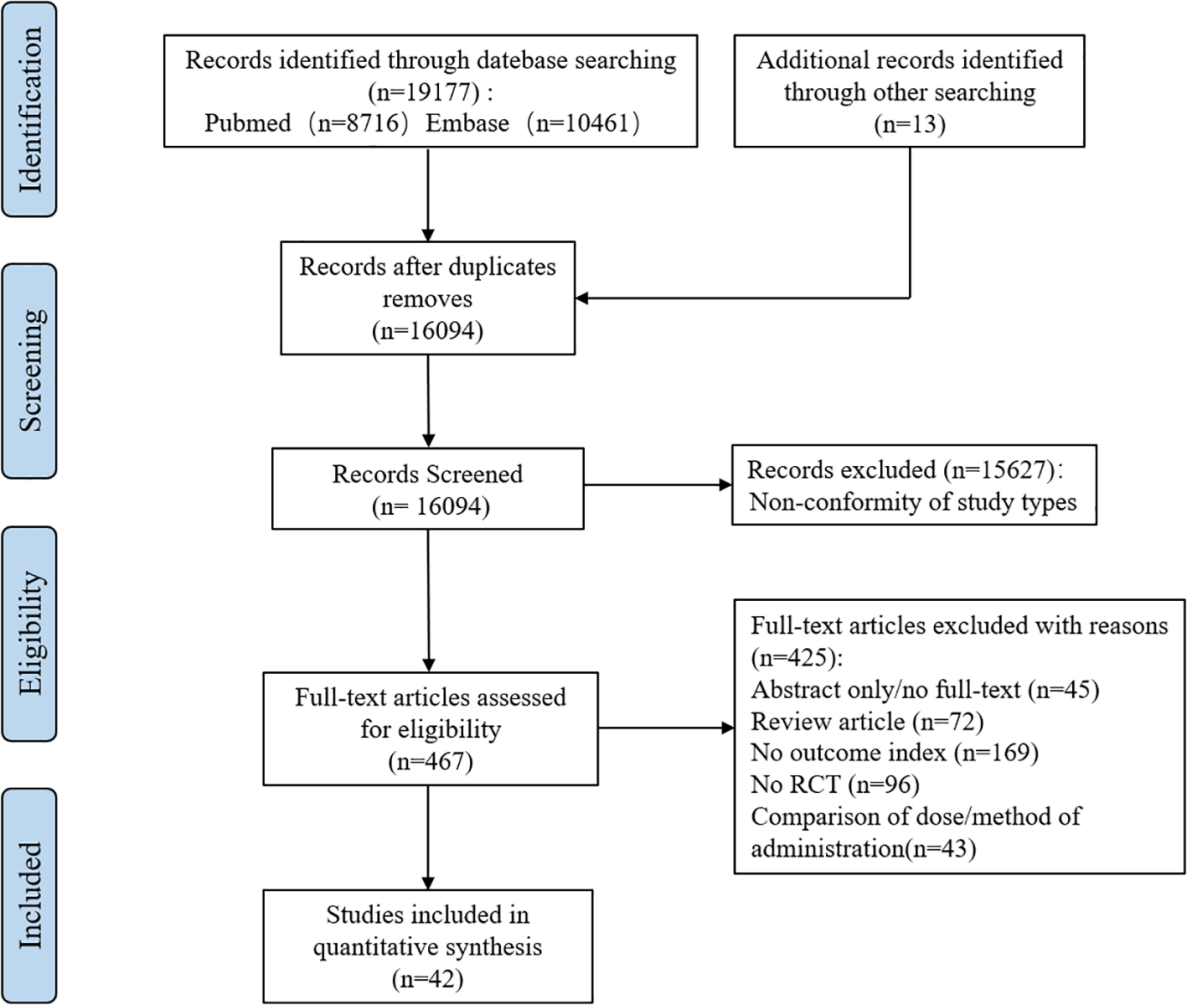
Flow chart of literature screening. (RCT, Randomized controlled trial).

We searched PubMed and Embase databases for a total of 19177 potentially relevant articles. After excluding 3083 duplicate articles, the full text of 467 articles was obtained by reading titles and abstracts. A full review of the literature resulted in the inclusion of 42 eligible studies that met the criteria (see **Figure 1**).

### Study characteristics

The main features of the included study (see **Table 1**) included a total of 8,382 subjects in 42 articles, all of whom were diagnosed with BV. In patients with BV, a total of eight drugs and a placebo were evaluated for monotherapy.

**Table 1:** Features of included studies (CR, cure rate; AE: adverse effects; NM, not mentioned).

Study	Study design	Interventions	Sample size	Administration mode and dosage	Treatment duration (days)	Outcomes
Larsson et al. (1990)	Double-blinded	Metronidazole	21	v.r 500mg tid	10	CR
		Placebo	21	N.A		
Buranawarodomkul et al. (1990)	Double-blinded	Metronidazole	93	p.o 1g qd	7	CR,AE
		Tinidazole	78	p.o 2g qd		
Bro (1990)	Double-blinded	Metronidazole	41	v.r 500mg qd	7	CR,AE
		Placebo	34	v.r 500mg qd		
Andres et al. (1992)	Double-blinded	Metronidazole	23	p.o 500mg bid	7	CR
		Clindamycin	23	v.r 2%5g qd		
Schmitt et al. (1992)	Double-blinded	Metronidazole	23	p.o 500mg bid	7	CR
		Clindamycin	25	p.o 2%5g qd		
Nayagam et al. (1992)	Clinical trial	Metronidazole	38	p.o 400mg bid	7	CR
		Ofloxacin	44	p.o 200mg bid		
Covino et al. (1993)	Double-blinded	Metronidazole	13	p.o 500mg bid	7	CR, AE
		Ofloxacin	14	p.o 300mg bid		
Stein et al. (1993)	Double-blinded	Clindamycin	57	v.r 2%5g qd	7	CR, AE
		Placebo	51	v.r 2%5g qd		
Fischbach et al. (1993)	Double-blinded	Metronidazole	110	p.o 500mg bid	7	CR
		Clindamycin	124	v.r 2%5g qd		
Boeke et al. (1993)	Clinical trial	Metronidazole	42	p.o 500mg bid	7	CR,AE
		Probiotics	37	v.r 100mg qd		
		Placebo	34	N.A		
Dhar et al. (1994)	Double-blinded	Clindamycin	23	v.r 2%20g qd	3	CR
		Placebo	21	v.r 2%20g qd		
Hill and Livengood (1994))	Double-blinded	Clindamycin	72	v.r 0.1%/1%/2% bid	5	CR
		Placebo	9	v.r 5g bid		
Livengood et al. (1994)	Double-blinded	Metronidazole	49	v.r. 0.75%5g bid	5	CR
		Placebo	41	v.r. 5g bid		
Ahmed-Jushuf et al. (1995)	Double-blinded	Clindamycin	107	v.r. 2%20g qd	3	CR, AE
		Placebo	114	v.r. 2%20g qd		
Saraçoğlu et al. (1998)	Open label	Ornidazole	55	p.o 500mg bid	5	CR
		Secnidazole	29	p.o 2g qd		
		Metronidazole	23	v.r.500mg bid	7	
Kurkinen-Räty et al. (2000)	Double-blinded	Clindamycin	51	v.r 5g qd	7	CR
		Placebo	50	v.r 5g qd		
Schwebke (2000)	Double-blinded	Metronidazole	28	v.r 5g qd	5	CR,AE
		Placebo	30	v.r 5g qd		
Kekki et al. (2001)	Double-blinded	Clindamycin	181	v.r 2% qd	7	CR
		Placebo	181	v.r 2% qd		
Voorspoels et al. (2002)	Double-blinded	Metronidazole	76	v.r 100/250/500mg	NM	CR
		Placebo	24	v.r		
Beigi et al. (2004)	Double-blinded	Metronidazole	49	v.r qd	5	CR
		Clindamycin	52	v.r 0.75% qd		
Anukam et al. (2006)	NM	Metronidazole	20	v.r 0.75% qd	5	CR
		Probiotics	20	v.r qd		
Livengood et al. (2007)	Double-blinded	Tinidazole	149	p.o 1/2g qd	2-5	CR
		Placebo	78	p.o qd	5	
Mastromarino et al. (2009)	Double-blinded	Probiotics	18	p.o qd	7	CR
		Placebo	16	p.o qd		
Zeng et al. (2010)	Double-blinded	Metronidazole	326	v.r 5g bid	5	CR
		Sucrose	108	v.r 5g bid		
		Placebo	109	v.r 5g bid		
Bohbot et al. (2010)	Double-blinded	Metronidazole	237	p.o 500mg bid	7	CR, AE
		Secnidazole	243	p.o 1g qd		
Schwebke and Desmond (2011)	Open label	Metronidazole	117	p.o 500mg bid	7	CR
		Tinidazole	232	p.o 0.5/1g bid		
Thulkar et al. (2012)	NM	Metronidazole	86	p.o 2g qd	7	CR
		Tinidazole	86	p.o 2g qd		
		Secnidazole	86	p.o 2g qd		
		Ornidazole	86	p.o 1.5g qd		
Hantoushzadeh et al. (2012)	Double-blinded	Probiotic	300	p.o 100g bid	7	CR
		Clindamycin	300	p.o 300mg bid		
Lamont et al. (2012)	Double-blinded	Clindamycin	171	v.r 100mg qd	3	CR
		Placebo	183	v.r 100mg	7	
Ling et al. (2013)	Clinical trial	Metronidazole	30	v.r 500mg qd	7	CR
		Probiotics	25	v.r qd	10	
Hillier et al. (2015)	Double-blinded	Secnidazole	126	p.o 1/2g	NM	CR
		Placebo	62	p.o 1g		
Schwebke et al. (2015)	Double-blinded	Metronidazole	250	v.r 5g bid	5	CR
		Placebo	237	v.r 5g bid		
Xiao et al. (2015)	Double-blinded	Metronidazole	207	v.r 10g	5	CR
		Sucrose	313	v.r 10g		
Hillier et al. (2017)	Double-blinded	Secnidazole	126	p.o 1/2g	NM	CR
		Placebo	62	p.o		
Tariq et al. (2017)	Double-blinded	Clindamycin	87	v.r	NM	CR
		Secnidazole	91	p.o 2g		
Schwebke et al. (2017)	Double-blinded	Secnidazole	107	v.r 2g	NM	CR,AE
		Placebo	57	v.r 2g		
Bohbot et al. (2018)	Double-blinded	Probiotics	39	v.r qd	14	CR, AE
		Placebo	39	v.r qd		
Khazaeian et al. (2018)	Double-blinded	Metronidazole	35	v.r 0.75%	14	CR
		Sucrose	35	v.r 9%		
Husain et al. (2020)	Double-blinded	Probiotics	123	p.o qd	NM	CR
		Placebo	115	p.o qd		
Pentikis et al. (2020)	Double-blinded	Secnidazole	169	p.o 1g2g	NM	CR,AE
		Placebo	119	p.o		
Cohen et al. (2020)	Double-blinded	Probiotics	152	p.o	7	CR, AE
		Placebo	76	p.o		
Ross et al. (2023)	Open label	Metronidazole	259	p.o 400mg bid	7	CR,AE
		Probiotics	259	v.r 4.5% qd		

### Deviation risk assessment

Of the 42 studies included, 39 were explicitly RCT studies. Three of the studies were non-RCT and double-blind, with a high risk of bias. Overall, all references included in this study had a low to moderate risk of bias (See **Supplementary Figures S1**, **S2**).

### Clinical cure rate

**Figure 2:**
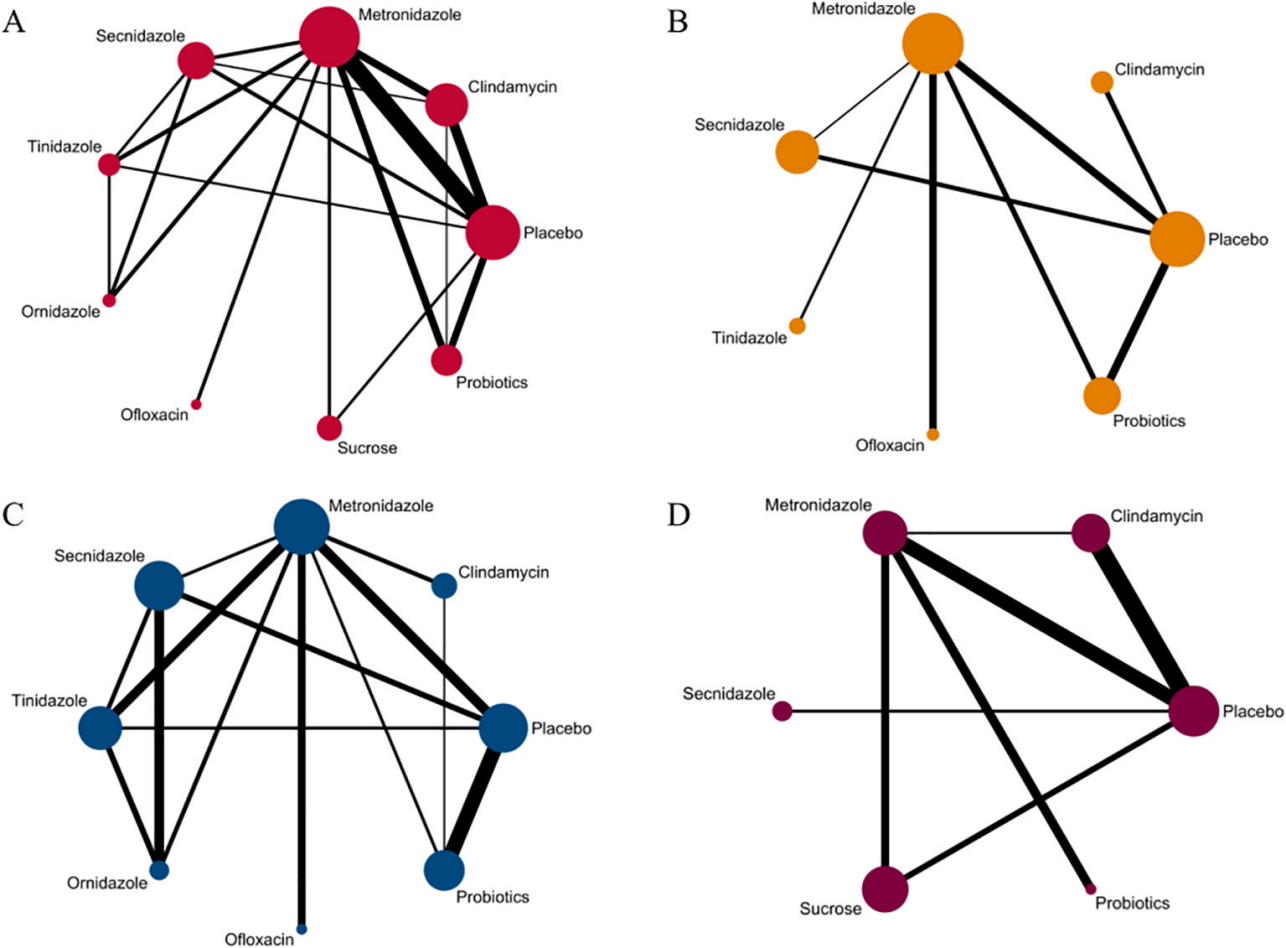
Network evidence graph. **(A)** Clinical cure rate. **(B)** incidence of adverse reactions. **(C)** Clinical cure rate of oral therapy. **(D)** Clinical cure rate of local vaginal administration. A graph of network evidence for the four study types, where the size of the nodes relates to the number of participants in that intervention type and the thickness of the line between interventions relates to the number of studies in that comparison.

Among the 42 included articles, the clinical cure rates of 8382 subjects were evaluated, of which 4686 were cured after treatment. The results of the direct meta-analysis are shown in **Supplementary Figure S3**, Direct meta-analysis results showed that ornidazole had a better clinical cure rate than Secnidazole (RR 1.22; 95%-CI 1.10-1.34).To further determine the direct and indirect comparative efficacy between the nine interventions, we conducted a network meta-analysis, and the network evidence graph is shown in **Figure 2A**. The results showed that clindamycin had a better clinical cure rate than placebo (RR 8.75; 95%-CI 3.74-20.48), the clinical cure rate of metronidazole was better than that of placebo (RR 6.21; 95%-CI 3.07-12.57), Secnidazole had a better clinical cure rate than placebo (RR 3.29; 95%-CI 1.26-8.59), the clinical cure rate of tinidazole was better than that of placebo (RR 8.83; 95%-CI 2.19-35.62), ornidazole had a better clinical cure rate than placebo (RR 15.13; 95%-CI 2.37-96.6), sucrose had a better clinical cure rate than placebo (RR 14.87; 95%-CI 3.04-72.67), the clinical success rate of probiotics was better than that of placebo (RR 3.89; 95%-CI 1.52-9.95), and the remainder were not found to be statistically significant (see **Table 2**).

**Table 2:** Clinical cure rates of various drug treatments.

Intervention	Placebo (RR, 95%-CI)	Clindamycin (RR, 95%-CI)	Metronidazole (RR, 95%-CI)	Secnidazole (RR, 95%-CI)	Tinidazole (RR, 95%-CI)	Ornidazole (RR, 95%-CI)	Ofloxacin (RR, 95%-CI)	Sucrose (RR, 95%-CI)	Probiotics (RR, 95%-CI)
Placebo	Placebo	**8.75 (3.74,20.48)**	**6.21 (3.07,12.57)**	**3.29 (1.26,8.59)**	**8.83 (2.19,35.62)**	**15.13 (2.37,96.60)**	3.33 (0.41,26.87)	**14.87 (3.04,72.67)**	**3.89 (1.52,9.95)**
Clindamycin	0.11 (0.05,0.27)	Clindamycin	0.71 (0.29,1.75)	0.38 (0.12,1.20)	1.01 (0.22,4.69)	1.73 (0.25,12.19)	0.38 (0.04,3.32)	1.70 (0.31,9.23)	0.44 (0.14,1.37)
Metronidazole	0.16 (0.08,0.33)	1.41 (0.57,3.47)	Metronidazole	0.53 (0.19,1.45)	1.42 (0.38,5.34)	2.44 (0.40,14.79)	0.54 (0.08,3.84)	2.39 (0.56,10.31)	0.63 (0.24,1.67)
Secnidazole	0.30 (0.12,0.80)	2.66 (0.83,8.51)	1.89 (0.69,5.18)	Secnidazole	2.69 (0.58,12.52)	4.60 (0.75,28.11)	1.01 (0.11,9.23)	4.52 (0.78,26.23)	1.18 (0.33,4.23)
Tinidazole	0.11 (0.03,0.46)	0.99 (0.21,4.61)	0.70 (0.19,2.64)	0.37 (0.08,1.74)	Tinidazole	1.71 (0.21,13.97)	0.38 (0.04,4.05)	1.68 (0.24,11.97)	0.44 (0.09,2.16)
Ornidazole	0.07 (0.01,0.42)	0.58 (0.08,4.08)	0.41 (0.07,2.49)	0.22 (0.04,1.33)	0.58 (0.07,4.75)	Ornidazole	0.22 (0.02,3.17)	0.98 (0.10,9.93)	0.26 (0.03,1.91)
Ofloxacin	0.30 (0.04,2.42)	2.62 (0.30,22.85)	1.86 (0.26,13.30)	0.99 (0.11,8.96)	2.65 (0.25,28.33)	4.54 (0.32,65.30)	Ofloxacin	4.46 (0.38,51.70)	1.17 (0.13,10.50)
Sucrose	0.07 (0.01,0.33)	0.59 (0.11,3.20)	0.42 (0.10,1.80)	0.22 (0.04,1.28)	0.59 (0.08,4.22)	1.02 (0.10,10.28)	0.22 (0.02,2.60)	Sucrose	0.26 (0.05,1.48)
Probiotics	0.26 (0.10,0.66)	2.25 (0.73,6.91)	1.60 (0.60,4.24)	0.84 (0.24,3.02)	2.27 (0.46,11.12)	3.89 (0.52,28.89)	0.86 (0.10,7.71)	3.82 (0.67,21.67)	Probiotics

RR, relative risk; 95% CI, 95% confidence intervals. Each column describes the relative risk of the 95% confidence interval, which is guaranteed to be statistically significant when the 95% confidence interval does not include 1 (highlighted in bold).

**Figure 3:**
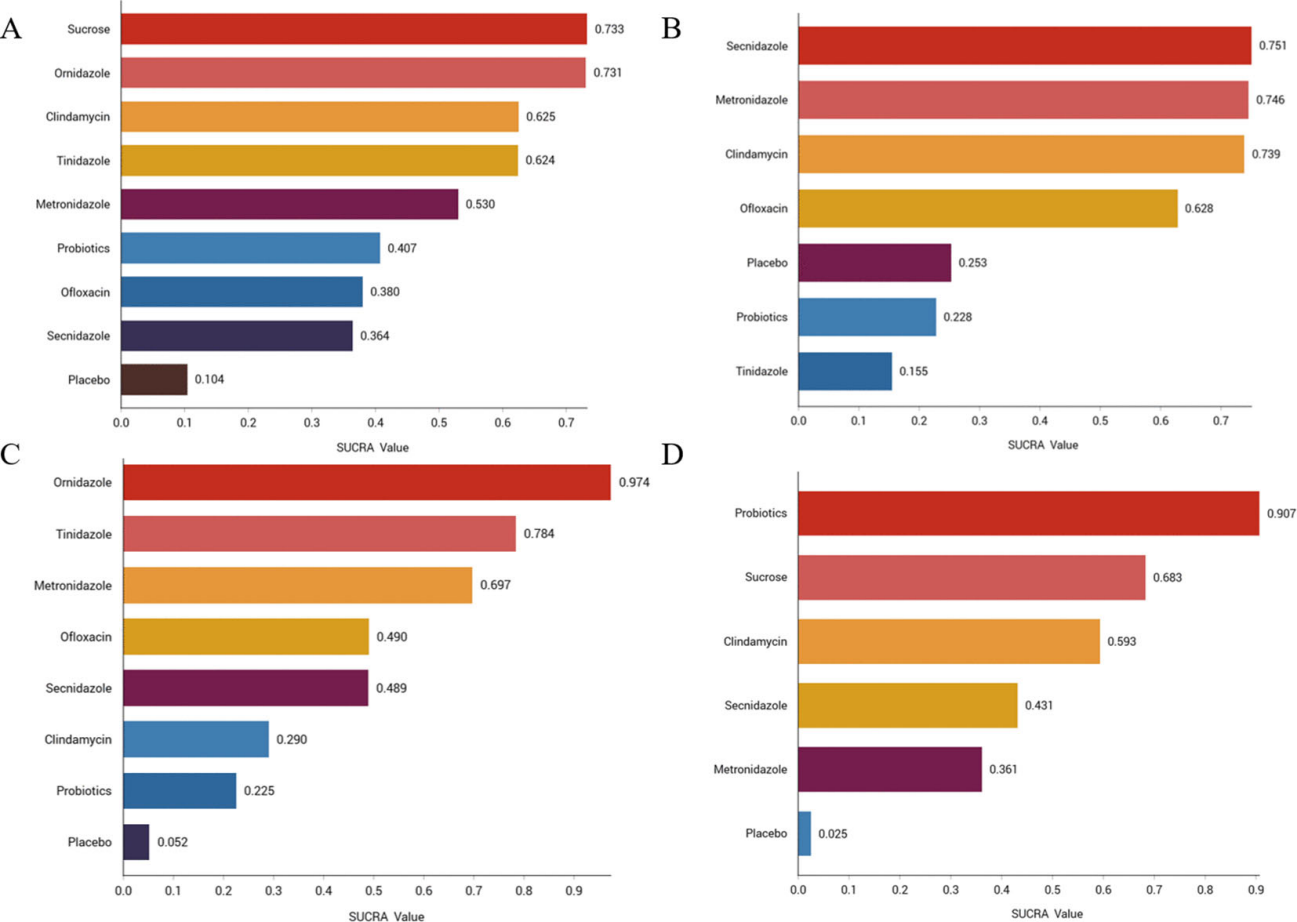
Efficacy and safety rankings of drugs used to treat BV **(A)** Clinical cure rate **(B)** Incidence of adverse reactions **(C)** Clinical cure rate of oral therapy **(D)** Clinical cure rate of local vaginal administration. SUCRA: Surface Under the Cumulative Ranking Curve.

We ranked the drugs according to their probability of clinical cure success (See **Figure 3A**). The results showed that sucrose (73.3%) ranked first, followed by ornidazole (73.1%), Clindamycin (62.5%), Tinidazole (62.4%), metronidazole (53%), probiotics (40.7%), ofloxacin (38%), Secnidazole (36.4%) and placebo (10.4%). See **Supplementary Figure S11** for the ranking cumulative probability of all treatment drugs.

### Adverse effects rate

Among the 42 included articles, a total of 13 articles evaluated 2524 subjects adverse reactions, of which 811 had adverse reactions. The results of the direct meta-analysis are shown in **Supplementary Figure S4**. To further determine the direct and indirect comparative efficacy of these six antibiotics, we conducted a network meta-analysis, and the network evidence graph is shown in **Figure 2B**. The evidence map, which directly or indirectly compared the therapeutic effects of six interventions, showed that metronidazole had a higher rate of adverse reactions than placebo (RR 2.14; 95%-CI 1.03-4.42), secnidazole had a higher adverse reaction rate than placebo (RR 2.20; 95%-CI 1.09-4.46), the adverse reaction rate of probiotics was lower than that of metronidazole (RR 0.44; 95%-CI 0.21-0.93), and no statistical significance was found in the comparison of the remaining drugs (See **Table 3**).

**Table 3:** Incidence of adverse reactions to various drug treatments.

Intervention	Placebo (RR, 95%-CI)	Clindamycin (RR, 95%-CI)	Metronidazole (RR, 95%-CI)	Secnidazole (RR, 95%-CI)	Tinidazole (RR, 95%-CI)	Ofloxacin (RR, 95%-CI)	Probiotics (RR, 95%-CI)
Placebo	Placebo	2.21 (0.91,5.38)	**2.14 (1.03,4.42)**	**2.20 (1.09,4.46)**	0.65 (0.14,2.96)	1.86 (0.53,6.60)	0.94 (0.48,1.82)
Clindamycin	0.45 (0.19,1.10)	Clindamycin	0.97 (0.31,3.05)	1.00 (0.32,3.10)	0.29 (0.05,1.71)	0.84 (0.18,3.96)	0.42 (0.14,1.28)
Metronidazole	0.47 (0.23,0.97)	1.04 (0.33,3.27)	Metronidazole	1.03 (0.48,2.20)	0.30 (0.08,1.15)	0.87 (0.31,2.48)	**0.44 (0.21,0.93)**
Secnidazole	0.45 (0.22,0.92)	1.00 (0.32,3.13)	0.97 (0.45,2.07)	Secnidazole	0.29 (0.06,1.37)	0.85 (0.23,3.07)	0.43 (0.18,1.02)
Tinidazole	1.54 (0.34,7.02)	3.40 (0.59,19.79)	3.29 (0.87,12.46)	3.39 (0.73,15.71)	Tinidazole	2.87 (0.53,15.60)	1.44 (0.31,6.65)
Ofloxacin	0.54 (0.15,1.90)	1.19 (0.25,5.57)	1.15 (0.40,3.26)	1.18 (0.33,4.29)	0.35 (0.06,1.90)	Ofloxacin	0.50 (0.14,1.80)
Probiotics	1.07 (0.55,2.07)	2.36 (0.78,7.16)	2.28 (1.08,4.83)	2.35 (0.98,5.64)	0.69 (0.15,3.20)	1.99 (0.55,7.14)	Probiotics

RR, relative risk,; 95% CI, 95% confidence intervals. Each column describes the relative risk of the 95% confidence interval, which is guaranteed to be statistically significant when the 95% confidence interval does not include 1 (highlighted in bold).

We ranked antibiotics according to their probability of adverse effects (See **Figure 3B**). The results showed that Secnidazole (75.1%) ranked first place, followed by metronidazole (74.6%), Clindamycin (73.9%), ofloxacin (62.8%), placebo (25.3%), probiotics (22.8%), tinidazole (22.8%). The ranking cumulative probability of all drug treatments is plotted in **Supplementary Figure S12**.

### Clinical cure rate of oral drugs in patients with BV

Among the 42 publications included, 19 evaluated the clinical cure rate of 3788 subjects with BV with oral drugs, of whom 2312 were successfully cured. To further determine the direct and indirect comparative efficacy of these eight interventions, we conducted a network meta-analysis, and the network evidence graph is shown in **Figure 2C**. The results of the direct meta-analysis are shown in **Supplementary Figure S5**, the clinical cure rate of ornidazole was better than Secnidazole (RR 1.23; 95%-CI 1.11-1.36). The evidence map, which directly or indirectly compared the therapeutic effects of eight interventions, showed that metronidazole had a better clinical cure rate than placebo (RR 7.06; 95%-CI 2.61-19.10, RR 4.03; 95%-CI 1.63-9.98), Secnidazole had a better clinical cure rate than placebo (RR 4.03; 95%-CI 1.63-9.98), tinidazole had a better clinical cure rate than placebo (RR 9.53; 95%-CI 2.80-189.59), ornidazole had a better clinical cure rate than placebo (RR 32.96; 95%-CI 5.73-189.59), the clinical cure rate of ornidazole was better than clindamycin (RR 16.08; 95%-CI 1.72-150.47), the clinical cure rate of probiotics was worse than that of metronidazole (RR 0.24; 95%-CI 0.08-0.77), ornidazole had a better clinical cure rate than Secnidazole (RR 8.17; 95%-CI 1.66-40.25), the clinical cure rate of probiotics was worse than tinidazole (RR 0.18; 95%-CI 0.04-0.72), the clinical cure rate of probiotics was worse than ornidazole (RR 0.05; 95%-CI 0.01-0.34), and no statistical significance was found in the comparison of the remaining antibiotics (See **Table 4**).

**Table 4:** Clinical cure rates of oral treatment of various drugs.

Intervention	Placebo (RR, 95%-CI)	Clindamycin (RR, 95%-CI)	Metronidazole (RR, 95%-CI)	Secnidazole (RR, 95%-CI)	Tinidazole (RR, 95%-CI)	Ornidazole (RR, 95%-CI)	Ofloxacin (RR, 95%-CI)	Probiotics (RR, 95%-CI)
Placebo	Placebo	2.05 (0.42,9.97)	**7.06 (2.61,19.10)**	**4.03 (1.63,9.98)**	**9.53 (2.80,32.49)**	**32.96 (5.73,189.59)**	4.09 (0.67,25.01)	1.71 (0.73,4.02)
Clindamycin	0.49 (0.10,2.37)	Clindamycin	3.44 (0.71,16.69)	1.97 (0.36,10.85)	4.65 (0.76,28.43)	**16.08 (1.72,150.47)**	2.00 (0.22,17.92)	0.83 (0.19,3.62)
Metronidazole	0.14 (0.05,0.38)	0.29 (0.06,1.41)	Metronidazole	0.57 (0.21,1.55)	1.35 (0.48,3.83)	4.67 (0.85,25.77)	0.58 (0.13,2.66)	**0.24 (0.08,0.77)**
Secnidazole	0.25 (0.10,0.61)	0.51 (0.09,2.80)	1.75 (0.65,4.75)	Secnidazole	2.36 (0.67,8.35)	**8.17 (1.66,40.25)**	1.02 (0.17,6.22)	0.42 (0.13,1.38)
Tinidazole	0.10 (0.03,0.36)	0.22 (0.04,1.32)	0.74 (0.26,2.10)	0.42 (0.12,1.49)	Tinidazole	3.46 (0.54,22.08)	0.43 (0.07,2.74)	**0.18 (0.04,0.72)**
Ornidazole	0.03 (0.01,0.17)	0.06 (0.01,0.58)	0.21 (0.04,1.18)	0.12 (0.02,0.60)	0.29 (0.05,1.85)	Ornidazole	0.12 (0.01,1.22)	**0.05 (0.01,0.34)**
Ofloxacin	0.24 (0.04,1.49)	0.50 (0.06,4.49)	1.72 (0.38,7.91)	0.98 (0.16,6.03)	2.33 (0.37,14.83)	8.05 (0.82,78.78)	Ofloxacin	0.42 (0.06,2.81)
Probiotics	0.59 (0.25,1.38)	1.20 (0.28,5.22)	4.13 (1.31,13.07)	2.36 (0.72,7.69)	5.58 (1.38,22.49)	19.29 (2.91,127.80)	2.40 (0.36,16.12)	Probiotics

RR, relative risk; 95% CI, 95% confidence intervals. Each column describes the relative risk of the 95% confidence interval, which is guaranteed to be statistically significant when the 95% confidence interval does not include 1 (highlighted in bold).

We ranked antibiotics according to their probability in terms of clinical cure rate (See **Figure 3C**). The results showed that ornidazole (97.4%) ranked the first place, followed by Tinidazole (78.4%), metronidazole (69.7%), ofloxacin (49%), Secnidazole (48.9%), clindamycin (29%), probiotics (22.5%) and placebo (5.2%). The ranking cumulative probability of all drug treatments is plotted in **Supplementary Figure S13**.

### Clinical cure rate of vaginal application of drugs in patients with BV

Among the 42 kinds of publications included, a total of 20 publications evaluated the clinical cure rate of 3808 cases of BV patients with vaginal application of drugs, of which 1953 cases were successfully cured. The results of the direct meta-analysis are shown in **Supplementary Figure S6**. The results showed that the clinical cure rate of sucrose was better than metronidazole (RR 1.12; 95%-CI 1.03-1.21), the clinical cure rate of metronidazole was worse than that of probiotics (RR 0.68; 95%-CI 0.52-0.88).To further determine the direct and indirect comparative efficacy of these six interventions, we conducted a network meta-analysis, and the network evidence graph is shown in **Figure 2D**. The evidence map, which directly or indirectly compared the therapeutic effects of six interventions, showed clindamycin had a better clinical cure rate than placebo (RR 8.40; 95%-CI 3.04-23.20), the clinical cure rate of metronidazole was better than that of placebo (RR 4.59; 95%-CI 1.75-12.06), sucrose had a better clinical cure rate than placebo (RR 11.42; 95%-CI 2.08-62.80), the clinical cure rate of probiotics was superior to placebo (RR 37.83; 95%-CI 3.53-405.69) and no statistical significance was found in the comparison of the remaining antibiotics (See **Table 5**).

**Table 5:** Clinical cure rate of local vaginal administration.

Intervention	Placebo (RR, 95%-CI)	Clindamycin (RR, 95%-CI)	Metronidazole (RR, 95%-CI)	Secnidazole (RR, 95%-CI)	Sucrose (RR, 95%-CI)	Probiotics (RR, 95%-CI)
Placebo	Placebo	**8.40 (3.04,23.20)**	**4.59 (1.75,12.06)**	4.77 (0.36,62.87)	**11.42 (2.08,62.80)**	**37.83 (3.53,405.69)**
Clindamycin	0.12 (0.04,0.33)	Clindamycin	0.55 (0.15,1.94)	0.57 (0.04,9.08)	1.36 (0.21,8.92)	4.50 (0.37,55.27)
Metronidazole	0.22 (0.08,0.57)	1.83 (0.52,6.48)	Metronidazole	1.04 (0.07,16.31)	2.49 (0.57,10.77)	8.24 (0.94,71.84)
Secnidazole	0.21 (0.02,2.77)	1.76 (0.11,28.18)	0.96 (0.06,15.13)	Secnidazole	2.40 (0.11,52.74)	7.94 (0.24,263.97)
Sucrose	0.09 (0.02,0.48)	0.74 (0.11,4.82)	0.40 (0.09,1.74)	0.42 (0.02,9.19)	Sucrose	3.31 (0.24,45.23)
Probiotics	0.03 (0.00,0.28)	0.22 (0.02,2.72)	0.12 (0.01,1.06)	0.13 (0.00,4.19)	0.30 (0.02,4.12)	Probiotics

Each column describes the relative risk of the 95% confidence interval, which is guaranteed to be statistically significant when the 95% confidence interval does not include 1 (highlighted in bold).

We ranked interventions according to their probability in terms of clinical cure rates (See **Figure 3D**). The results showed that probiotics (90.7%) ranked first, followed by sucrose (68.3%), clindamycin (59.3%), Seknidazole (43.1%), metronidazole (36.1%), and placebo (2.5%). The ranking cumulative probability of all drug treatments is plotted in **Supplementary Figure S14**.

### Publishes bias and inconsistency evaluations

By observing the symmetry of the funnel plot to detect publication bias, the funnel plot in this study was basically in a symmetric distribution, and no significant publication bias was found in all results, and 11 points were distributed outside the 95%CI, indicating the possible influence of small sample size. (See **Supplementary Figures S15**–**S18**).

## Discussion

The NMA aims to evaluate the effectiveness and safety of monotherapy with various drugs for the treatment of BV. A NMA of clinical cure rates and adverse event rates for all 42 included studies was performed using random-effects models, and subgroup analyses were performed for two different routes of administration, oral and vaginal. To our knowledge, this is the first study to analyze a single drug for patients diagnosed with BV (Muñoz-Barreno et al., 2021). The study by Alison et al. focused on evaluating the effectiveness of antibiotics or antibiotic and probiotic combination therapy, not a comparison between single drugs (Muñoz-Barreno et al., 2021). The study by Tan et al. also only compared the effectiveness of metronidazole or metronidazole in combination with probiotics and was not a network meta-analysis (Tan et al., 2017).

In our analysis of clinical cure rates for BV, we found that most of the drugs had higher clinical cure rates than placebo, and there was no significant difference in the single comparison between the drugs. However, a further evaluation of 42 studies allowed us to identify certain therapies that worked better. SUCRA value is a method used to evaluate the effectiveness of different interventions, a higher SUCRA value indicates a better treatment outcome. According to the SUCRA value, sucrose had the highest cure rate, followed by ornidazole and clindamycin. Sucrose is the main source of glucose for the normal microbiota, reduces the pH of the growth environment of pathogenic bacteria, and effectively inhibits the growth of pathogenic bacteria (Lidbeck and Nord, 1993). Current studies have also shown that sucrose gel is more effective than metronidazole gel and is an effective alternative therapy (Zeng et al., 2010). However, more clinical drug comparisons, different treatment times, and long-term follow-up investigations are needed to further confirm these results. Ornidazole, a second-generation 5-nitroimidazole drug, is currently being used as a treatment for BV, and this drug has a spectrum of activity against BV-associated anaerobic microorganisms (Zhao et al., 2023). Clindamycin, which is recommended by the CDC for various first-line treatments of BV in women, has also shown good clinical cure rates (Workowski and Bolan, 2015).

In the analysis of the incidence of adverse reactions in BV, we found that the incidence of adverse reactions of metronidazole was higher than that of placebo, the main adverse effects of metronidazole include gastrointestinal symptoms, increased vaginal discharge, and itching. And that of secnidazole was higher than that of placebo, the incidence of adverse reactions to probiotics was lower than that of metronidazole (RR 0.44; 95%-CI 0.21-0.93), which is consistent with the results of relevant clinical trials (Boeke et al., 1993; Ross et al., 2023). According to the SUCRA value, we found that the lowest incidence of adverse reactions was tinidazole, followed by probiotics and placebo. The safety of probiotics has been reported in the literature, but in the literature included in this study, the evaluation of adverse reactions was less. In future studies, more adverse reactions of single drug therapy should be investigated.

This study provides more meaningful clues as we conducted a subgroup analysis of different dosing modes in cure rates and found that there was a significant difference in efficacy between oral and vaginal administration. Ornidazole had the highest probability of oral administration, followed by tinidazole and metronidazole. The results of the network meta also showed that the clinical cure rate of ornidazole was better than clindamycin (RR 16.08; 95%-CI 1.72-150.47), ornidazole had a better clinical cure rate than Secnidazole (RR 8.17; 95%-CI 1.66-40.25), the clinical cure rate of probiotics was worse than ornidazole (RR 0.05; 95%-CI 0.01-0.34), indicating that oral ornidazole has a better effect than clindamycin and probiotics, the two traditional therapeutic drugs, but there are few studies on the side effects of Ornidazole, and it is unknown whether it will cause serious adverse reactions. Metronidazole, the drug of choice for the treatment of BV, has the lowest half-life of 7.9-8.8h (Mattila et al., 1983). Other nitroimidazole drugs newly appearing in the market have different half-lives, among which Ornidazole and tinidazole have longer half-lives of 14-14.7h and 14.1-16.8h, respectively (Martin et al., 1990; Gillis et al., 1996). This makes it have a better effect on the treatment process, and a longer half-life has a better control effect on pathogenic bacteria, which is the main reason for choosing oral administration. Probiotics had the highest probability of cure with vaginal administration, followed by sucrose and clindamycin, and there was no crossover with oral drugs. Probiotic therapy has a significant effect on BV treatment. When probiotics are applied to the vagina, probiotics quickly colonize the vagina, occupying the colonization site of pathogenic bacteria, effectively reducing pathogenic bacteria, and improving the recovery rate of normal bacteria of BV (Liu and Yi, 2022).In addition, probiotics can produce a variety of antibacterial substances and lactic acid, thus inhibiting the growth of pathogenic bacteria and stimulating the immune system through competitive adhesion to achieve therapeutic effects (Ling et al., 2013).

For the timing of the visit for the test of cure, most of the literature included in this study evaluated patients 21-30 days after the start of treatment, and a few evaluated patients 2-3 months. Differences in the time of assessment also lead to a bias in the final clinical cure rate, which generally decreases the longer the visit. As the visit time increases, the patients also face an increased risk of secondary infections, such as douching during life, sexual transmission, or failure of initial treatment to completely eradicate all the microbes that cause bacterial vaginitis, all of which can lead to reinfection of BV.

In this study, there are still some limitations: first, the sample size of individual studies is limited; Second, different selection criteria are used in individual studies. Then there was heterogeneity between studies in terms of patient populations, probiotic strains, and outcome follow-up. In the end, individual studies gave different doses. In addition, there is currently insufficient data to demonstrate significant differences between the efficacy of other drugs to treat BV, and more high-quality clinical studies are needed to verify this. And in future studies, the side effects of drug treatment should also receive more attention.

## Conclusion

This systematic review and network meta-analysis is the first study to analyze a drug for the treatment of BV, and the results suggest that ornidazole may be an effective option for the treatment of BV, while sucrose and probiotics are potential BV treatments, and more clinical trial studies are needed in the future to verify this idea.
